# The effects of physical exercise on college students’ pro-social behavior: the chain mediating role of sense of meaning in life and subjective well-being

**DOI:** 10.3389/fpsyg.2025.1604700

**Published:** 2025-06-18

**Authors:** Yong Jiang, Tianqi Bian

**Affiliations:** School of Physical Education, Liaoning Normal University, Dalian, China

**Keywords:** physical exercise, pro-social behavior, sense of meaning in life, subjective well-being, chain intermediation

## Abstract

To explore the relationship between the sense of meaning of life and subjective well-being in college students’ physical exercise and pro-social behavior. A scale was used to measure 626 college students, and SPSS27.0 and Process4.1 were used for statistical analysis. Results (1) college students’ physical exercise, sense of meaning in life, subjective well-being and pro-social behavior were positively correlated. (2) Physical exercise significantly and positively predicted college students’ pro-social behavior (*β* = 0.284, *p* < 0.001); the effect value of the indirect effect path 1 was 0.0028 (physical exercise → sense of meaning in life → pro-social behavior), which accounted for 31.11% of the total effect; the effect value of the indirect effect path 2 was 0.0007 (physical exercise → sense of meaning in life → pro-social behavior). The effect value of indirect effect path 1 is 0.0028 (physical exercise → sense of meaning in life → subjective well-being → pro-social behavior) which accounts for 31.11% of the total effect; the effect value of indirect effect path 2 is 0.0007 (physical exercise → subjective well-being → pro-social behavior) which accounts for 7.78% of the total effect; and the effect value of indirect effect path 3 is 0.0013 (physical exercise → sense of meaning in life → subjective well-being → pro-social behavior) which accounts for 14.44% of the total effect. Therefore, physical exercise has a direct effect on the pro-social behavior of college students, and can also indirectly affect pro-social behavior through the sense of meaning of life and subjective well-being.

## 1 Introduction

The United Nations Educational, Scientific and Cultural Organization (UNESCO) mentioned in the Policy Guidelines for Quality Physical Education that quality physical education should integrate physical, mental and socio-emotional learning, and develop students’ pro-social qualities such as a sense of responsibility and respect for others through structured sports programs. As a core group of talents for national development, the cultivation of pro-social behavior of college students is not only related to the improvement of individual social adaptability, but also involves the deeper needs of social harmony and civilization progress (Zhang et al., 2021). Pro-social behavior refers to the behaviors that individuals show in social interactions, which are beneficial to others, groups or the society, and such behaviors are expected and encouraged by the society, and the actors do not expect to obtain direct personal benefits from them, but rather, out of voluntariness and self-awareness, they bring benefits to others or the society (Eisenberg and Fabes, 1998). Physical exercise, as a physical activity with a high level of daily participation among college students, not only enhances physical fitness, but also has been shown to be closely associated with psychological health and social skill development (Reigal et al., 2020; Di Bartolomeo and Papa, 2019). Among them, psychological sense of meaning in life and subjective well-being are the key mediating variables connecting physical exercise and pro-social behavior. Based on this, this study explores the relationship between physical exercise, sense of meaning in life, subjective well-being and pro-social behavior of college students. To reveal the unique advantages of physical exercise in enhancing the pro-social behavior of college students, to provide targeted suggestions for improving the pro-social behavior of college students, and to promote the overall development of college students’ physical and mental health.

## 2 Research hypotheses

### 2.1 Physical exercise and the predictive effects of pro-social behavior in college students

Physical exercise is an activity in which an individual performs physical exercises according to his or her own needs through various physical means for the purpose of enhancing physical fitness, improving health, promoting physical and mental development, and improving physical functioning (Xi, 2004). A large number of studies have shown that there is a significant positive association between physical exercise and pro-social behavior (Xiao and Qiu, 2023; Wan et al., 2021). First, social learning theory states that individuals can develop pro-social behaviors by observing the behaviors of others in the process of participating in physical exercise and by using the reinforcement mechanism of behavioral effects (Majed et al., 2022). Secondly, physical activity driven by participation in physical exercise not only enhances individual empathy, promotes the sense of collective participation, enhances interpersonal trust, and thus strengthens pro-social behavior, but also improves mental health, forms a more positive emotional state, and thus creates favorable conditions for the implementation of pro-social behavior (Bahmani et al., 2019). As a result, during physical activity, individuals are able to deal with competition and cooperation in a coordinated manner, thus demonstrating pro-social behaviors such as humility, respect, and solidarity (Goldstein and Iso-Ahola, 2006). Finally, the study by Moeijes et al. (2019) further confirmed that individuals who are actively involved in physical activity tend to show more significant pro-social behavioral tendencies and reduce aggressive behavior. The above study suggests that there may be an association between physical exercise and pro-social behavior. Hypothesis H1 is proposed: Physical exercise can positively predict college students’ pro-social behavior.

### 2.2 Mediating effects of sense of meaning in life

The sense of meaning in life refers to an individual’s perception of the meaning and value of life that he or she possesses at the present moment, and the experience and search for the meaning of life in the process of striving to realize self-worth (Tian et al., 2023). Exercise psychology research has found that college students’ physical exercise is closely related to the sense of meaning in life, and strengthening physical exercise is conducive to improving the level of college students’ sense of meaning in life (Zeng and Zhu, 2021). The sense of meaning in life construction model proposed by Park and Folkman (1997) has an important theoretical influence, which systematically describes the process of individuals’ perception and construction of meaning in life in different contexts. When individuals engage in physical exercise, they can actively construct situational meaning through physical practice and integrate it as part of the overall sense of meaning in life, and the enhancement of this sense of meaning is further promoted through the intrinsic motivation mechanism in the self-determination theory, which prompts individuals to link their self-worth with social needs, thus enhancing the initiative of pro-social behavior. Therefore, the proposed model not only provides theoretical guidance for college students to explore the goals and values of life and enhance the sense of meaning in life, but also lays an important theoretical foundation for an in-depth study of the intrinsic association between the sense of meaning in life and physical exercise (Liu, 2024). In addition, individuals with a higher sense of meaning in life are more likely to integrate into social groups and are more inclined to take practical actions for the collective good, demonstrating more realistic behaviors (Sun, 2018). Relevant studies have pointed out that there is a significant positive correlation between the intensity of an individual’s intrinsic perception of the meaning of life and his or her goal setting and persistent pursuit. Specifically, when individuals face the severe test of life stress, a higher sense of meaning in life can stimulate their ability and confidence to cope with difficulties and manage challenges, thus significantly promoting the improvement and enhancement of their pro-social behavior (Zhang, 2022; Jafary et al., 2011). Based on the above research, physical exercise affects the acquisition of the sense of meaning in life, and the sense of meaning in life affects the enhancement of college students’ pro-social behavior ability, so the hypothesis H2 is proposed: the sense of meaning in life plays a mediator role in the effect of physical exercise on college students’ pro-social behavior.

### 2.3 Mediating effects of subjective well-being

Subjective well-being refers to an individual’s perception of the overall quality of life according to his or her own criteria, and is a subjective experience and attitude that includes an emotional component and a cognitive component (Diener, 1984). A study by Qiao and Fan (2020) pointed out that physical exercise is effective in enhancing the level of self-esteem, life satisfaction, and health benefits of participants by providing them with pleasurable and hedonistic experiences, thus significantly enhancing subjective well-being. Research by Claus (2017) further confirmed that physical exercise can help individuals achieve a lasting and stable state of well-being (Tov and Diener, 2008). In addition, a positive emotional state can significantly contribute to an individual’s pro-social behavior performance (Lyubomirsky et al., 2005). Aknin et al. (2012) found that there is a significant positive feedback loop between pro-social behavior and well-being: recalling past pro-social behaviors (e.g., donating to others) can significantly increase an individual’s sense of well-being, and this enhanced sense of well-being further increases the likelihood that the individual will continue to engage in pro-social behavior. Based on the above research, physical exercise affects the acquisition of subjective well-being, and subjective well-being affects the enhancement of college students’ ability to engage in pro-social behavior, therefore, Hypothesis H3 is proposed: subjective well-being plays a mediating role in the effect of physical exercise on college students’ pro-social behavior.

### 2.4 Chain mediation of sense of meaning in life and subjective well-being

In the study of the influence of physical exercise on college students’ pro-social behavior, both the sense of meaning in life and subjective well-being play a simple mediating effect, and there is an interrelationship between the sense of meaning in life and subjective well-being. Studies by scholars such as Li and Xie (2015) and Xiao et al. (2010) have shown that the increase in the level of sense of meaning in life of college students has a significant positive effect on the improvement of subjective well-being. Individuals realize the construction of a sense of meaning in life through physical exercise (Park and Folkman, 1997), and the enhancement of this sense of meaning is transformed into subjective well-being through two paths: on the one hand, exercise behaviors compatible with the meaning of life can stimulate intrinsic motivation (Jin et al., 2016), prompting individuals to obtain a stable sense of well-being in the continuous pursuit of meaning (Wong, 2012); on the other hand, a sense of meaning in life formed in physical exercise enhances an individual’s positive perception of life as a whole (Li and Xie, 2015; Xiao et al., 2010), thus indirectly improving subjective well-being. The enhancement of subjective well-being further influences individuals’ pro-social behavior through the mechanism of “positive feedback loop,” which makes individuals show more pro-social behavior (Aknin et al., 2012). It can be seen that individuals with a clear meaning of life are more inclined to look at the problems in life from a positive perspective, improve the cognitive evaluation of life satisfaction, and enhance the subjective well-being of individuals; whereas individuals without a clear goal of life tend to have cognitive and emotional dysfunction in the face of intractable internal and external pressures, thus falling into the state of imbalance and helplessness, and inhibiting the expression of pro-social behavior (Wang et al., 2018). Specifically, physical exercise firstly enhances the individual’s sense of meaning in life through physical practice and situational meaning construction, the enhancement of sense of meaning in life then promotes subjective well-being through intrinsic motivation stimulation and positive cognitive reinforcement, and the enhancement of subjective well-being will ultimately enhance the individual’s tendency of pro-social behavior through the drive of positive emotions and the optimization of social interactions. Therefore, Hypothesis H4 is proposed: the sense of meaning in life and subjective well-being may play a chain mediating role in the effect of physical exercise on pro-social behavior of college students.

**Figure 1:**
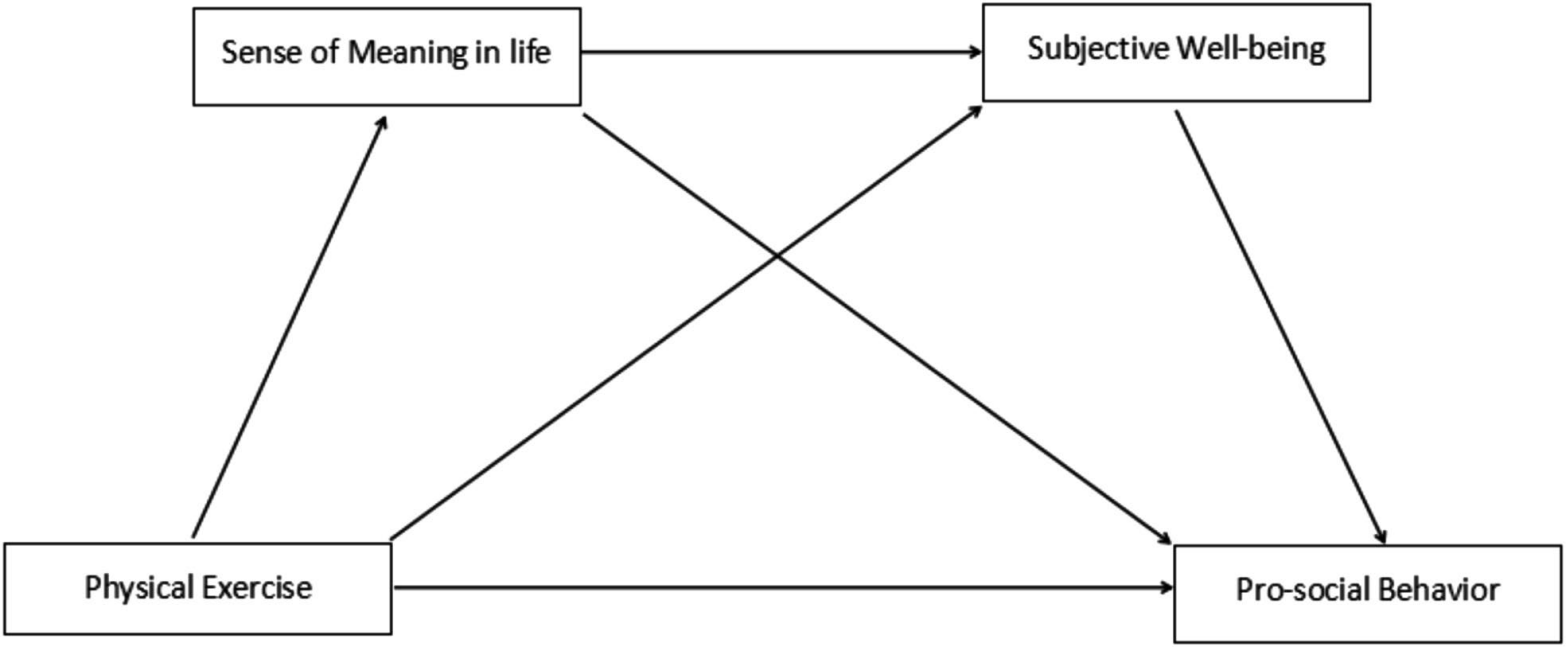
Diagram of the hypothetical model.

As a result, the research hypothesis model was constructed as shown in Figure 1.

## 3 Research objectives and methods

### 3.1 Research object

The research object of this paper is the effect of physical exercise on college students’ pro-social behavior and the mediating role of sense of meaning in life and subjective well-being. In order to improve the representativeness of the sample to the whole and to ensure the coverage of college student groups at different stages of study, this study used stratified random sampling based on grade level to select college students of four grades as the subjects, and distributed 630 questionnaires in total. Among them, four invalid questionnaires were excluded, including extreme responses, incomplete responses, and responses that did not comply with the time limit, with extreme responses being those that had exactly the same options and were overly biased. 626 valid questionnaires were recovered, with an effective recovery rate of 99.37%, and the sample size conformed to the standard. The demographics of the participants are detailed in the Supplementary Table.

### 3.2 Research tools

#### 3.2.1 Physical activity rating scale

The Physical Activity Rating Scale revised by Liang (1994) was used with the aim of examining the indicators of intensity, time, and frequency of subjects’ participation in physical exercise in the past month. Each index was scored on a 5-point Likert scale, with physical exercise = intensity × (time-1) × frequency, and was categorized according to the total score as small exercise ≤ 19 points, medium exercise = 20 to 42 points, and large exercise ≥ 43 to 100 points, with a total of three grades, which were used as the evaluation indexes of the subjects’ physical exercise.

#### 3.2.2 Pro-social behavior scale

The Pro-Social Behavior Scale compiled by Carlo and Randall (2002) and revised by Yu et al. was used with the aim of assessing the pro-social behavior of college students, which consists of 26 items with six dimensions, namely, Openness, Anonymity, Altruism, Compliance, Emotionality, and Urgency, and is assessed on a 5-point scale, with higher scores indicating a stronger tendency towards pro-social behavior. In the present study, the Cronbach’s alpha coefficient for the retest was 0.965.

#### 3.2.3 Sense of meaning in life scale

The Sense of Meaning in Life Scale written by Steger et al. (2006) and revised by Wang et al. was used to assess the sense of meaning in life of college students. This scale has ten questions, which include two dimensions about having a sense of meaning and seeking a sense of meaning, with five questions each within each dimension, and the original questionnaire adopts a seven-point scoring method, and in order to facilitate the analysis, the present study adopted Liket’s five-point scoring system for the assessment, and the score The higher the score, the stronger the sense of meaning in life. In this study, the Cronbach’s alpha coefficient for the retest was 0.874.

#### 3.2.4 Subjective well-being scale

The subjective well-being scale developed by Zhang et al. (2007) was used to assess the subjective well-being of college students, which includes two levels in its structure, self-satisfaction and environmental satisfaction, the former is divided into 4 dimensions, namely, friendship, family, academics, and freedom, and the latter is divided into 2 dimensions, namely, school and environment, with a total of 36 questions in 6 dimensions. The original questionnaire used a 7-point scoring system, and in order to facilitate the analysis, in this study, Liket’s 5-point scoring system was used for assessment, with higher scores indicating greater subjective well-being. In this study, the retested Cronbach’s alpha coefficient was 0.948.

### 3.3 Statistical analysis

Data entry and organization of the questionnaires were carried out using Excel to screen out invalid questionnaires. SPSS27.0 software and PROCESS plug-in were used to statistically analyze the collected data. Harman’s one-way method was used to test for the presence of common method bias, and Pearson’s correlation analysis was used to explore the relationship between the main variables. In addition, the chain mediation effect was tested with the help of Bootstrap program in PROCESS plug-in.

### 3.4 Information collection

Information was collected in the form of questionnaire distribution, which consisted of the Physical Activity Rating Scale, Pro-Social Behavior Scale, Sense of Meaning in Life Scale, and Subjective Wellbeing Scale, facing the college student population in the Dalian and Chongqing regions, and online questionnaire distribution was used; the questionnaire was distributed on January 15, 2025, and was collected on February 10, 2025. The design of the study followed the guiding principles and provisions of the Declaration of Helsinki and was approved by the Ethics Committee of Liaoning Normal University (LL2025018); all participants were adults who obtained verbal informed consent prior to participation and were ensured that they fully understood the study objectives, procedures and potential risks. The results of valid questionnaires returned were 626, 325 (51.9%) male and 301 (48.1%) female; in terms of grade, 338 (54%) were freshmen, 269 (43%) were sophomores, 10 (1.6%) were juniors, and 9 (1.4%) were seniors.

## 4 Research results and analysis

### 4.1 Common method bias test

Due to the influence of the measurement environment, questionnaire instruction and context, the questionnaire survey to obtain data may have the problem of common method bias. The Harman one-way test was conducted on the measurement data, and the unrotated principal component analysis of physical exercise, pro-social behavior, sense of meaning in life, and subjective well-being was conducted by using SPSS27.0, and the results showed that there were a total of 11 factors with eigenroots greater than 1, and the first factor explanation rate was 37.678%, which was less than the critical value of 40%, indicating that the data of the present study did not suffer from the problem of common method bias.

### 4.2 Descriptive statistics

Descriptive statistical analysis showed (Table 1) that there were 626 college students in the sample, of which 325 (51.9%) were male and 301 (48.1%) were female. This indicates that the number of male and female students participating in the survey is relatively equal. In terms of grade distribution, first-year students were the most numerous with 338 students, accounting for 54%, second-year students had 269 students, accounting for 43%, third-year students had 10 students, accounting for 1.6%, and fourth-year students had 9 students, accounting for 1.4%.

**Table 1:** Demographic analysis.

Causality	Form	Quantities	Percentage
Gender	Male student	325	51.9%
	Female student	301	48.1%
Grade	Girst-year university student	338	54%
	Second-year university student	269	43%
	Third-year university student	10	1.6%
	Fourth-year university student	9	1.4%

In addition, the independent samples *t*-test was used to analyze (Table 2) the differences in physical exercise, pro-social behavior, sense of meaning in life and subjective well-being among college students of different genders. The results showed that boys scored slightly higher (*M* = 30.308, SD = 21.229) than girls (*M* = 29.249, SD = 21.130) on the amount of physical exercise, but did not reach the level of significance (*t* = 0.625, *p* = 0.532). Differences in pro-social behavior were demonstrated by slightly higher scores for girls (*M* = 3.753, SD = 0.634) than boys (*M* = 3.726, SD = 0.700), which again did not show a significant difference (*t* = −0.510, *p* = 0.611). In terms of psychological variables, girls’ score of sense of meaning in life (*M* = 3.632, SD = 0.647) was close (*t* = −0.450, *p* = 0.653) to boys’ (*M* = 3.609, SD = 0.667) while boys’ sense of subjective well-being (*M* = 3.676, SD = 0.595) was higher than that of girls’ (*M* = 3.584, SD = 0.544) and did not show significant difference (*t* = 2.017, *p* = 0.44).

**Table 2:** Independent samples *t*-test.

	Gender	*N*	*M* ± SD	*t*	*p*
Physical exercise	Male	325	30.308 ± 21.229	0.625	0.532
	Female	301	29.249 ± 21.130		
Pro-social behavior	Male	325	3.726 ± 0.700	−0.510	0.611
	Female	301	3.753 ± 0.634		
Sense of meaning in life	Male	325	3.609 ± 0.667	−0.450	0.653
	Female	301	3.632 ± 0.647		
Subjective well-being	Male	325	3.676 ± 0.595	2.017	0.44
	Female	301	3.584 ± 0.544		

ANOVA one-way analysis (Table 3) was used to analyze the differences in physical exercise, pro-social behavior, sense of meaning in life and subjective well-being among college students of different grades. The results showed that there was a significant difference in the amount of physical exercise among college students of different grades (*F* = 3.677, *p* < 0.05). Specifically, the amount of physical exercise among juniors (*M* = 43.400, SD = 27.224) and seniors (*M* = 47.444, SD = 35.472) was significantly higher than that of freshmen (*M* = 28.976, SD = 20.370) and sophomores (*M* = 29.736, SD = 21.044), and a *post hoc* test showed (Table 4) that the difference in mean values between the juniors and freshman year mean difference was 14.424, and the mean difference between juniors and freshmen was 18.468. However, the grade level differences in pro-social behavior (*F* = 0.816, *p* = 0.485), sense of meaning in life (*F* = 1.709, *p* = 0.164), and subjective well-being (*F* = 2.003, *p* = 0.112) did not reach the level of significance.

**Table 3:** ANOVA one-way analysis of variance.

Relevant variable	Grade	*M* ± SD	*F*	*p*
Physical exercise	1 (338)	28.976 ± 20.370	3.677	*p* < 0.05
	2 (269)	29.736 ± 21.044		
	3 (10)	43.400 ± 27.224		
	4 (9)	47.444 ± 35.472		
Pro-social behavior	1 (338)	3.749 ± 0.664	0.816	0.485
	2 (269)	3.710 ± 0.670		
	3 (10)	3.973 ± 0.734		
	4 (9)	3.919 ± 0.785		
Sense of meaning in life	1 (338)	3.621 ± 0.660	1.709	0.164
	2 (269)	3.596 ± 0.653		
	3 (10)	3.930 ± 0.706		
	4 (9)	3.967 ± 0.507		
Subjective well-being	1 (338)	3.644 ± 0.579	2.003	0.112
	2 (269)	3.596 ± 0.563		
	3 (10)	3.897 ± 0.594		
	4 (9)	3.941 ± 0.506		

**Table 4:** Multiple comparisons of physical exercise among college students at different grade levels.

Grade	First-year university student (28.976)	Second-year university student (29.736)	Third-year university student (43.400)
Fourth-year university student (47.444)	−18.468	−17.708	−4.044
Third-year university student (43.400)	−14.424	−13.664	
Second-year university student (29.736)	−0.760		

### 4.3 Correlation analysis of variables

Through Person correlation analysis of the data on physical exercise, pro-social behavior, sense of meaning in life and subjective well-being (Table 5) showed that physical exercise was significantly and positively correlated with pro-social behavior (*r* = 0.281, *p* < 0.01), and also significantly and positively correlated with sense of meaning in life (*r* = 0.202, *p* < 0.01) and subjective well-being (*r* = 0.203, *p* < 0.01) correlated. The correlation between sense of meaning in life and subjective well-being was also significant (*r* = 0.661, *p* < 0.01), and both were significantly and positively correlated with pro-social behavior (sense of meaning in life: *r* = 0.665, *p* < 0.01; subjective well-being: *r* = 0.618, *p* < 0.01).

**Table 5:** Person correlation analysis.

	Physical exercise	Pro-social behavior	Sense of meaning in life	Subjective well-being
Physical exercise	1			
Pro-social behavior	0.281^**^	1		
Sense of meaning in life	0.202^**^	0.665^**^	1	
Subjective well-being	0.203^**^	0.618^**^	0.661^**^	1

** Significant correlation at the 0.01 level (two-tailed).

### 4.4 Mediation effect test

The chain mediating effects of sense of meaning in life and subjective well-being between physical exercise and pro-social behavior were tested (Table 6). The steps were as follows: in the first step, using pro-social behavior as the dependent variable, gender and grade were entered to control for the possible effects of these variables on pro-social behavior. In the second step, physical exercise was entered to establish a regression model to examine the total effect of physical exercise on pro-social behavior after controlling for the variables, and it was found that physical exercise significantly and positively predicted pro-social behavior (*β* = 0.284, *p* < 0.001), which indicated that research hypothesis H1 was valid. In the third step, the sense of meaning in life and subjective well-being variables were sequentially added to the model to test whether they were mediating variables between physical exercise and pro-social behavior and whether there was a chain mediating effect, and it was found that physical exercise was able to significantly and positively predict pro-social behavior (*β* = 0.135, *p* < 0.001), the sense of meaning in life was able to positively predict pro-social behavior (*β* = 0.435, *p* < 0.001), and subjective well-being was able to positively predict pro-social behavior (*β* = 0.307, *p* < 0.001). Also in the test, it was found that physical exercise significantly positively predicted sense of meaning in life (*β* = 0.201, *p* < 0.001) and subjective well-being (*β* = 0.070, *p* < 0.05), and sense of meaning in life positively predicted subjective well-being (*β* = 0.649, *p* < 0.001). As seen from the above regression coefficients, there was a significant chain mediation between physical exercise and pro-social behavior for sense of meaning in life and subjective well-being, proving that research hypotheses H2 and H3 were valid.

**Table 6:** Regression analysis of variable relationships.

Equation of regression	Variable of prediction	Overall fit index			Significance of regression coefficients		
Result variable		*R*	*R* ^2^	*F*	*β*	*t*	*p*
Sense of meaning in life	Physical exercise	0.204	0.042	8.991	0.201	5.095	0.000^**^
	Gender				0.022	0.563	0.574
	Grade				0.017	0.421	0.674
Subjective well-being	Physical exercise	0.671	0.451	127.327	0.070	2.306	0.021^*^
	Sense of meaning in life				0.649	21.361	0.000^**^
	Gender				−0.090	−3.012	0.002
	Grade				−0.006	−0.215	0.830
Pro-social behavior	Physical exercise	0.720	0.518	133.060	0.135	4.681	0.000^**^
	Sense of meaning in life				0.435	11.588	0.000^**^
	Subjective well-being				0.307	8.167	0.000^**^
	Gender				0.042	1.492	0.136
	Grade				−0.028	−1.007	0.315
Pro-social behavior	Physical exercise	0.283	0.080	18.054	0.284	7.339	0.000^**^
	Gender				0.028	0.738	0.461
	Grade				−0.020	−0.509	0.611

**p < 0.001, *p < 0.05.

Bootstrap test was used to repeat the sampling 5,000 times to test the mediating effect of sense of meaning in life and subjective well-being between physical exercise and pro-social behavior as well as the confidence intervals, respectively (Table 7). The results showed that physical exercise produced a total effect value of 0.0090 on pro-social behavior, and the 95% confidence intervals for the mediating effects of sense of meaning in life and subjective well-being did not contain 0 (LLCL = 0.0066, ULCL = 0.0114), which indicated that the total effect of physical exercise on pro-social behavior as well as the mediating effects of the two variables were significant. The value of the direct effect of physical exercise on pro-social behavior is 0.0042 (direct path), Bootstrap 95% confidence interval does not contain 0 (LLCL = 0.0025, ULCL = 0.0060), which indicates that there is a significant direct effect of physical exercise on pro-social behavior, and the value of the effect is 46.67% of the total effect, as shown in Table 7.

**Table 7:** Intermediary effect size analysis.

Effect	Trails	Effect value	Standard error	LLCL	ULCL	Effect ratio
Total effect		0.0090	0.0012	0.0066	0.0114	100%
Direct effect	Direct path	0.0042	0.0009	0.0025	0.0060	46.67%
Total indirect effect		0.0047	0.0009	0.0029	0.0065	52.23%
Indirect effect	Ind1	0.0028	0.0006	0.0015	0.0040	31.11%
	Ind2	0.0007	0.0003	0.0001	0.0014	7.78%
	Ind3	0.0013	0.0004	0.0006	0.0020	14.44%

It can be seen that physical exercise predicts pro-social behavior, and that sense of meaning in life and subjective well-being play an indirect mediating effect in it (there are three paths), with a total indirect effect value of 0.0047, Bootstrap 95% confidence interval does not contain 0 (LLCL = 0.0029, ULCL = 0.0065), which accounts for 52.23% of the total effect. Among them, the first mediating effect path: physical exercise → sense of meaning in life → pro-social behavior (Path 1), with an indirect effect value of 0.0028, accounting for 31.11% of the total effect; the second path: physical exercise → subjective well-being → pro-social behavior (Path 2), with an indirect effect value of 0.0007, accounting for 7.78% of the total effect; and the third chain mediating effect path: physical exercise → sense of meaning in life → subjective well-being → pro-social behavior (path 3), with an indirect effect value of 0.0013, accounting for 14.44% of the total effect, indicating that research hypothesis H4 is valid.

**Figure 2:**
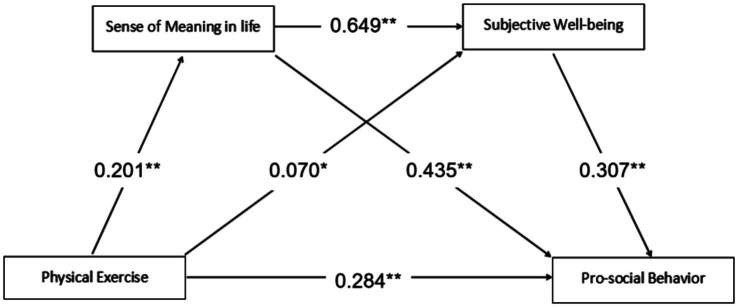
Intermediary model diagram.

Based on the above findings, the chain mediation model was derived as shown in Figure 2.

## 5 Discussion

### 5.1 Physical exercise has a significant positive effect on pro-social behavior among college students

The results of the study showed that the total effect value of physical exercise on college students’ pro-social behavior was 0.0090, of which the direct effect accounted for 46.67% (*β* = 0.284, *p* < 0.001), indicating that there is a correlation between the two, and that physical exercise positively predicts and directly promotes college students’ pro-social behavior, which is a result consistent with the existing studies (Xiao and Qiu, 2023; Wan et al., 2021; Majed et al., 2022; Bahmani et al., 2019; Goldstein and Iso-Ahola, 2006; Moeijes et al., 2019). Male students showed higher levels of physical exercise, but female students were better than male students in pro-social behavior, which may be caused by the differences in social expectations, psychological mechanisms and behavioral motives between men and women, but it does not affect the fact that physical exercise directly promotes pro-social behavior among college students. Physical exercise not only improves individual physical and mental health, but also enhances the sense of teamwork and collective belonging, thus motivating college students to show more pro-social behaviors in social interactions. Due to the special characteristics of college students, school sports show unique advantages in cultivating college students’ pro-social behavior. By integrating on-campus and off-campus educational resources, carrying out a variety of extracurricular practical activities, or adopting innovative teaching modes such as project-based learning and service learning, it can effectively enhance college students’ self-efficacy, self-management ability and emotional regulation, thus further promoting the development of pro-social behavior (Dong and Zhu, 2020).

### 5.2 Mediating effects of sense of meaning in life between physical exercise and college students’ pro-social behavior

The study shows that the indirect effect value of path 1 (physical exercise → sense of meaning in life → pro-social behavior) is 0.0028, accounting for 31.11% of the total indirect effect, indicating that the sense of meaning in life plays a mediating role in physical exercise and college students’ pro-social behavior, and that physical exercise not only directly affects the development of college students’ pro-social behavior, but also indirectly by positively affecting the sense of meaning in life, which indirectly indirectly by positively influencing the pro-social behavior of college students. Physical exercise can effectively improve individuals’ self-confidence and self-esteem, enhance their sense of independence and self-control ability, and relieve their stress and regulate their emotions. In the process of physical exercise, individuals activate the active construction of meaning in life through the goals and challenges of physical practice, and this kind of embodied action enables individuals to transfer the self-breakthrough experience of exercise to the whole of life, forming the cognition of creating value through active action, thus enhancing the sense of meaning in life. The enhancement of the sense of meaning in life further promotes individuals to connect their self-worth with social needs through the mechanism of intrinsic motivation in self-determination theory, thus enhancing the initiative of pro-social behavior (Liu et al., 2020; Zhang et al., 2010; Das, 1998). Moreover, the enhanced sense of meaning in life makes individuals more inclined to pay attention to the external world, accurately differentiate the experience of self and others, and deeply understand the needs of others, which promotes individuals to implement altruistic pro-social behavior (Chen et al., 2007; Zhang and Li, 2018).

### 5.3 The mediating effect of subjective well-being between physical exercise and pro-social behavior among college students

The results of data analysis showed that the indirect effect value of path 2 (physical exercise → subjective well-being → pro-social behavior) was 0.0007, accounting for 7.78% of the total indirect effect, indicating that subjective well-being also plays a certain mediating role in physical exercise and college students’ pro-social behavior, and that physical exercise positively predicts subjective well-being (*β* = 0.070, *p* < 0.05), and subjective well-being also positively predicted college students’ pro-social behavior (*β* = 0.307, *p* < 0.001), further confirming the previous hypotheses. Research has shown (Ma and Yan, 2010) that moderate physical exercise can effectively alleviate negative emotions such as depression, dependence and anxiety in college students, thus significantly enhancing their subjective well-being. In contrast, college students with higher subjective well-being were more likely to exhibit pro-social behavioral tendencies. Specifically, physical exercise enhances subjective well-being through both physiological and psychological pathways: on the one hand, the improvement of physical function brought about by regular exercise can significantly increase the self-esteem level of individuals; on the other hand, the social interaction and goal achievement experience in physical exercise further strengthens the individual’s perception of life satisfaction. This subjective well-being enhancement, in turn, will ultimately improve the individual’s pro-social behavior tendency through the drive of positive emotions and the optimization of social interactions. When college students have a high level of self-esteem and hold a high level of satisfaction with life, their psychological state tends to be more full and optimistic. This positive psychological state not only enhances their sensitivity to the needs and plight of others, but also stimulates their intrinsic motivation based on gratitude and sharing, making them practice pro-social behaviors more actively, thus forming a virtuous cycle of personal happiness and social harmony in the campus and social environment.

### 5.4 Chain-mediated effects of sense of meaning in life and subjective well-being between physical exercise and pro-social behavior among college students

Upon analysis of the findings, the indirect effect value of path 3 (physical exercise → sense of meaning in life → subjective well-being → pro-social behavior) was 0.0013, which accounted for 14.44% of the total indirect effect, indicating that the sense of meaning in life not only directly affects pro-social behavior (*β* = 0.435, *p* < 0.001), but also further reinforces the process by enhancing subjective well-being (*β* = 0.649, *p* < 0.001). On the one hand, the sense of meaning in life acquired by college students through physical exercise is a key factor influencing their subjective well-being, and studies have shown that there is a stable and systematic correlation between the two, which confirms that the sense of meaning in life plays a significant role in enhancing individual subjective well-being; on the other hand, physical exercise can synergistically promote the development of college students’ pro-social behaviors from both internal and external dimensions. Individuals transform their exercise experience into an active construction of meaning in life through physical practices such as setting exercise goals and overcoming exercise challenges during physical exercise, based on the sense of meaning construction model, and form a cognitive framework for action to create value, thereby enhancing the sense of meaning in life; the enhancement of the sense of meaning in life, on the one hand, stimulates intrinsic motivation through exercise behaviors that are compatible with the sense of meaning, so that the individual obtains a stable sense of well-being experience in the continuous pursuit of meaning, and on the other hand, it enhances subjective well-being by increasing the individual’s positive perceptions of life as a whole; the enhancement of subjective well-being broadens the scope of individual cognition and enhances individual empathy through the extended construct theory of positive emotions, forming a positive feedback loop between subjective well-being and pro-social behavior, and driving individuals to proactively implement pro-social behavior through intrinsic motivation. The chain mediation proposed in this study reveals that physical exercise not only positively predicts college students’ pro-social behavior through the separate mediating roles of sense of meaning in life and subjective well-being, but also may jointly influence college students’ pro-social behavior through the chain mediation of sense of meaning in life and subjective well-being, proving the value and significance of the two mediating variables for the cultivation of college students’ pro-social behavior, which is conducive to a more comprehensive understanding of the internal mechanism of physical exercise’s influence on college students’ pro-social behavior.

## 6 Conclusion

Through data analysis, the following conclusions can be drawn: (1) There is a significant correlation between physical exercise, pro-social behavior, sense of meaning in life, and subjective well-being. (2) Physical exercise positively predicts pro-social behavior in college students. (3) Physical exercise indirectly positively predicts college students’ pro-social behavior through the sense of meaning in life and subjective well-being. This mediating effect consists of three pathways: the individual mediating effect of sense of meaning in life and subjective well-being and the chain mediating effect of sense of meaning in life—subjective well-being.

## 7 Limitations and future directions

This study reveals the promotional effect of physical exercise on college students’ pro-social behavior and its intrinsic mechanism of action, but there are still some limitations that need to be further improved in future studies. First, the sample mainly focused on college students in Liaoning and Chongqing, and the sample coverage needs to be expanded in the future to enhance the generalizability of the conclusions; second, the cross-sectional study design is difficult to clarify the causal relationship between variables, and the temporal sequence of mediating pathways can be further verified in the follow-up through longitudinal tracking or experimental interventions; finally, the measurement of pro-social behavior relied on self-reporting method, which may have a social approval bias, and the future can combine with Behavioral observation or peer evaluation and other multi-source data to improve the validity; In addition, the article did not examine the moderating role of individual traits (e.g., personality, values) or environmental factors (e.g., family support, campus culture), and a more complex integration model can be constructed in the future. Future studies can further explore the interaction mechanisms between physical exercise and other positive psychological variables (e.g., empathy, mental toughness), and develop comprehensive intervention programs integrating physical exercise, sense of meaning shaping and well-being enhancement to provide practical guidance for mental health education in colleges and universities.

## Data Availability

The original contributions presented in the study are included in the article/supplementary material, further inquiries can be directed to the corresponding author.
